# The Impact of Sleep Deprivation on Brain Networks in Response to Social Evaluation Tasks

**DOI:** 10.3390/brainsci13081122

**Published:** 2023-07-25

**Authors:** Yiqi Mi, Huimin Duan, Ziye Xu, Xu Lei

**Affiliations:** 1 Sleep and NeuroImaging Center, Faculty of Psychology, Southwest University, Chongqing 400715, China; 2 Key Laboratory of Cognition and Personality, Ministry of Education, Chongqing 400715, China

**Keywords:** sleep deprivation, social feedback, social evaluation task, default mode network, self-referential processing, cognitive control

## Abstract

Sleep loss may lead to negative bias during social interaction. In the current study, we conducted a revised social evaluation task experiment to investigate how sleep deprivation influences the self-referential and cognitive processes of social feedback. The experiment consisted of a first impression task and a social feedback task. Seventy-eight participants completed the first impression task and were divided into normal and poor sleep groups. The results of an independent samples *t*-test showed that participants who slept worse were less likely to socialize with others but did not evaluate others as less attractive. Afterward, 22 of the participants from the first impression task were recruited to complete the social feedback task during functional magnetic resonance imaging (fMRI) on the mornings following two different sleep conditions at night: one night of normal sleep and one night of sleep deprivation. The results of this within-subject design study showed that participants who experienced the latter condition showed increased activation within the default mode network (i.e. superior parietal lobule, precuneus, inferior parietal lobule, inferior temporal gyrus, and medial frontal gyrus) and anterior cingulate cortex (ACC) and stronger negative insula functional connectivity (FC) with the precuneus to negative feedback than positive feedback. The altered activation and behavioral pattern may indicate a negative bias for social cues. However, stronger negative coupling may indicate stronger cognitive control, which may protect against potential damage to self-concept. Our study suggested that sleep impairs most social functions, but may protect against impairment of important ones, such as self-concept.

## 1. Introduction

Human beings are born to interact. When entering universities, social skills were required among college students to manage new relationships. However, college studentsare vulnerable to sleep disturbances [1]. And sleep-deprived individuals were found to be negative-oriented during social interaction [2]. They were always in a state of hyperarousal when facing social threats, preferred larger social distance, and felt lonelier [3,4]. Sleep deprivation was also followed by decreased social activities and willingness to socialize [5,6]. 

Social evaluation is one of the most important cues during social interaction. This cue indicates our social standing [7] and is especially salient when we join new groups [8]. Positive feedback prompts social behaviors, motivates us to pursue desirable interpersonal relationships, and develops confidence [9]. Social rejection reminds us to take measures to maintain interpersonal relationships [10]. It is usually followed by unpleasant feelings due to actual and potential damage to self-value and social networks [11]. What is more, in addition to processing others’ feedback, our evaluation of others also affects social behaviors. Study participants were found to be less likely to socialize with those who look tired and less attractive [12,13].

Neural circuits involved in affective functions mediate approach and avoidance responses. Affective circuity implicated in social feedback processing includes the ventral anterior cingulate, amygdala, anterior insula, striatum, and hypothalamus [9,11,14]. Moreover, regions involved in mentalizing others and self (e.g., temporal–parietal junction (TPJ), precuneus, posterior cingulate cortex (PCC)) were also found to be activated during the social evaluation processing [7,9]. The insula has been identified as a core region to integrate the function of emotion and cognition [15,16]. Insula–frontoparietal region connectivity was found to be involved in cognitive control and plays a critical role in social evaluation processing [15]. 

However, few studies have investigated whether sleep loss leads to dysfunction in the processing of social feedback. In one study, in terms of evaluating others, participants were found to consider others as less trustworthy and attractive after 24 h of sleep deprivation [13]. They were also evaluated as looking more exhausted and lonelier [4,12]. It was also reported that people were less likely to socialize with those who look tired [12]. According to these findings, we conjectured that people who were chronically sleep deprived may rate others’ faces as less attractive, just as those who experienced acute sleep deprivation did and this lower judgment of others’ facial attractiveness may be associated with a lower willingness to socialize.

As regards the processing of others’ social feedback, we were especially interested in emotional and neural responses following negative evaluation. Sleep loss has been identified as being closely related to greater feelings of hurt during social rejection [17,18]. Gilbert et al. (2015) conducted a 7-day longitudinal study to investigate how sleep was related to social rejection [17]. In their study, the amount of sleep was measured by wristwatch actigraphy and sleep diaries. Participants needed to report their daily rejection events and subjective feelings. The authors found a negative relationship between the amount of sleep and social rejection. In addition to emotional response, Gordon et al. (2019) measured cardiac responses when experiencing negative evaluation [18]. They found shorter interbeat intervals (IBI) in participants with sleep disturbances, which indicated greater physical arousal. As previous studies largely focused on self-reporting and psychophysiological response, it is important to examine whether the brain exhibits different patterns of activation and functional connectivity (FC) between normal sleep and sleep deprivation condition.

The current study aimed to investigate the relationship between sleep loss and social evaluation. A behavioral between-subject design task and a neuroimaging within-subject design task were included to discuss this question. Both emotional response and brain functions were measured.

## 2. Materials and Methods

**Figure 1:**
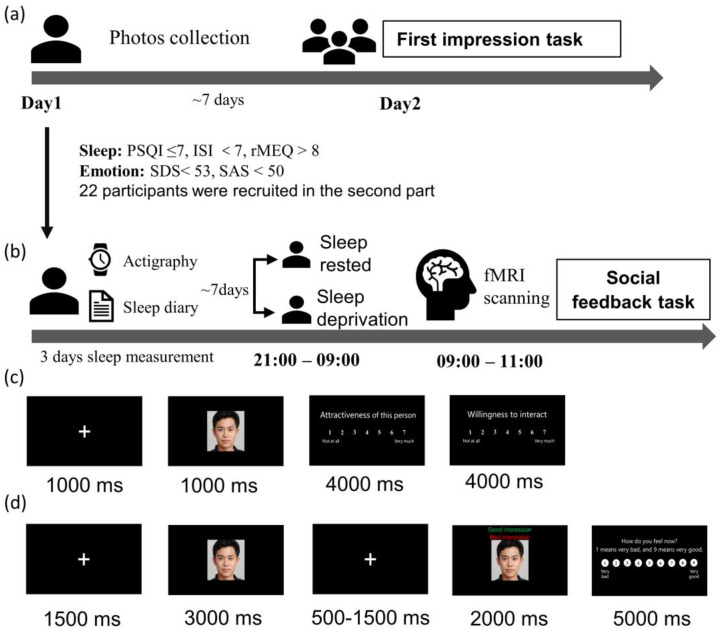
Study procedure (**a**) the first impression task of the revised social evaluation task experiment; (**b**) social feedback task; (**c**) first impression task, the participants were required to rate their desire to socialize with these strangers and the strangers’ facial attractiveness on a scale of 1–7 (1 = not at all, 7 = very much); (**d**) social feedback task, participants were required to rate their feelings from 1 (very negative) to 9 (very positive) after receiving pieces of feedback.

A revised social evaluation task experiment was conducted in the current study, consisting of a first impression task and a social feedback task. The scheme for the study can be found in Figure 1. The first impression task (Figure 1a) was a behavioral experiment using an independent sample between-subject design. It attempted to determine if chronic sleep loss affects how we assess others. Those who completed the first part and were without any sleep and emotional disorders were eligible to participate in the social feedback task (Figure 1b), which had a within-subject design. During this task, they received positive and negative feedback during functional magnetic resonance imaging (fMRI) scanning. The current revised version was based on the original experiment by Somerville et al. (2006) and modified based on the experiment by Guyer et al. (2012) [9,14].

The study was conducted in accordance with the Declaration of Helsinki and approved by the local human studies committee of Southwest University (code: H22035). A letter of consent was obtained from the participants on arrival. 

### 2.1. Participants

In the first impression task, seventy-nine college students (57 women; 19.59 ± 1.45 years of age) were enrolled through online advertisements. Only students aged 18 to 28 were included in our study. None of the participants had been diagnosed with mental disorders. 

After the task, they were divided into a normal sleep group and a poor sleep group based on Pittsburgh Sleep Quality Index (PSQI) and Insomnia Severity Index (ISI) score. At this stage, one of the 79 participants was excluded due to data corruption. A total of 37 of 78 were normal sleepers (PSQI ≤ 7, ISI ≤ 7) [19], while 41 of 78 were poor sleepers (PSQI > 7 or ISI > 7). Levels of social anxiety and rejection sensitivity were measured after the task.

In the social feedback task, 22 of the participants who finished the first impression task (14 women; 19.05 ± 1.19 years of age) were kept on to undergo an fMRI scan. All 22 were right-handed, had normal and corrected to normal vision, and had no contraindication for MRI. They were also free from sleep or emotional disorders. Inclusion criteria were determined using a series of questionnaires, including PSQI, ISI, reduced Morning–Evening Questionnaire (rMEQ), Self-Rating Depression Scale (SDS), and Self-Rating Anxiety Scale (SAS). The inclusion criteria were PSQI ≤ 7, ISI ≤ 7, rMEQ > 8, SAS < 50, and SDS < 53. A total of 2 of the 22 participants who completed the task were excluded due to excessive head movement during the scan, thus, 20 (12 women; 19.10 ± 1.22 years of age) were retained. 

### 2.2. Experimental Design and Procedure

#### 2.2.1. Stimuli

All the facial images were collected at Southwest University and Chongqing Jiaotong University and consisted of 80 female and 80 male subjects. Photos were taken using a CANON 6D, 1024 × 1024, 24-bit depth camera. All the images were used in the first impression task; however, photos of participants and their acquaintances were excluded in the following analysis, these pictures being identified based on self-reporting. A total of 60 of 160 images were included in the data analysis. For the social feedback task, photos of participants and their acquaintances were excluded, which led to 128 of 160 being used. These were divided into two parts, one of which was used after a night of normal sleep and the other of which was used after a night of sleep deprivation. There were no differences in facial appearance (*t* = −1.00, *p* = 0.315).

#### 2.2.2. First Impression Task

In the first impression task, participants were photographed and asked to give a first impression of the faces of people unfamiliar to them (Figure 1c). When participants first arrived, they were told that the study’s purpose was to investigate college students’ first impressions of their peers. We took a photo of each participant during this visit. At least one week after the photos were taken, the participants returned to the lab. They were told that they needed to rate their desire to socialize with a set of strangers (i.e., how much they want to interact with those strangers) and the facial attractiveness of the strangers on a scale of 1–7 (1 = not at all, 7 = very much). The photos displayed in the task were those that we took one week earlier. Finally, they were told that others would evaluate them in the same way. In this study, 79 participants were recruited. One was later excluded due to missing data. 

#### 2.2.3. Sleep Manipulation 

Participants who met the screening criteria went through fMRI scanning in a counterbalanced order: one scan after a night of normal sleep (SN) and one after 24 h of sleep deprivation (SD). The two conditions were separated by at least one week. Participants were asked to wear awristwatch actigraphyand complete sleep diaries for 72 h prior to the study. These measures were taken to ensure that they had no sleep debt before scanning. Participants refrained from alcohol and caffeine during the study.

In the sleep-deprived session, participants were required to stay awake for 24 h. They arrived at the laboratory at 9:00 p.m. and were monitored by experimenters and surveillance cameras. During the night, they engaged in a limited set of activities, including watching movies, reading, walking, and surfing the internet. In the sleep-rested session, participants slept in the college dormitories and were monitored by wristwatch actigraphy, allowing for more naturalistic sleep. In both cases, they arrived at the MRI scan center the next day at 9:00 a.m. and completed a social feedback task inside the MRI scanner.

#### 2.2.4. Social Feedback Task

The second task used a rapid, event-related design which comprise 2 runs of 32 trials (64 trials in total) and took about 18 min. During scanning, participants received positive or negative feedback. Half the pieces of feedback were positive and half were negative, and they were presented in a random order. Participants were told that these pieces of feedback were collected during the first impression task. In truth, they were created by the experimenters. Each picture was displayed for 3 s without feedback. After 0.5–1.5 s, the word ‘good impression’ or ‘bad impression’ was displayed at the bottom of the photograph. Subsequently, a rating scale was displayed for 5 s, and participants rated their emotional feelings toward the feedback from 1 to 9 (1 = very negative, 5 = neutral, 9 = very positive). 

Only data from participants who believed the feedback was actually from others were included in the analysis. No one had any doubt about the feedback that they received. At the end of the experiment, they were told that the feedback they had received was, in fact, fabricated.

#### 2.2.5. Image Acquisition

Blood oxygenation level-dependent contrast functional images were acquired with an echo-planar imaging (EPI) T2*-weighted sequence using a Siemens 3 Tesla MRI scanner with a 64-channel head coil. Each image volume consisted of 62 slices (TR/TE = 2000/30 ms, FOV = 224 × 224 mm^2^, flip angle = 90°, acquisition matrix = 112 × 112, thickness/gap = 2/0.3 mm, in-plane resolution = 2.0 × 2.0 mm^2^). Each run consisted of the acquisition of 270 successive brain volumes. A high-resolution T1-weighted anatomical image was acquired using a 3D GR/IR sequence (TR/TE = 2530/2.98 ms, FOV = 256 × 256 mm^2^, flip angle = 7°, acquisition matrix = 224 × 224, reconstruction matrix = 448 × 448 thickness/gap = 1/0 mm, in-plane resolution = 0.5 × 0.5 mm^2^, slices = 192) at the end of scanning. Field map scanning was also performed before BOLD image acquisition for susceptibility correction during subsequent preprocessing.

### 2.3. Behavioral Statistical Analysis

All analyses were performed using IBM SPSS Statistics 26.0. An independent samples *t*-test was used to compare differences in social evaluation behaviors between the normal sleep group and the poor sleep group. For each participant, scores for their desire to socialize with a set of strangers and the strangers’ facial attractiveness were averaged across the task. In the social feedback task, a repeated measures two-way analysis was conducted to investigate the common effect of sleep conditions and feedback type on emotional arousal triggered by social feedback.

### 2.4. Imaging Statistical Analysis

#### 2.4.1. Data Preprocessing

The imaging data were processed and analyzed using SPM12 (https://www.fil.ion.ucl.ac.uk/spm, accessed on 13 January 2020). Images were spatially realigned to the first image to correct for head movements and field maps were used for susceptibility correction when performing realignment. Images were then slice-scan-time-corrected and normalized to the Montreal Neurological Institute template and smoothed using a 6 mm full-width-at-half-maximum Gaussian Kernel.

#### 2.4.2. Whole-Brain Analysis

Trials with excessive head motion (over 2 mm absolute displacement in any direction or Framewise Displacement(FD) > 0.9) were de-weighted using a regressor of no interest in the GLM [20]. Two participants were dropped from the analysis due to excessive head movements in over 10% of time points. Contrast was created at the first level focusing on negative vs. positive feedback. Six motion parameters indicating rotation and translation were added as regressors. The resulting contrast was then taken through to the second level, using a paired *t*-test (SN vs. SD) to asses group-level effects. 

We applied the statistical thresholds for the analysis of brain activation (*p* < 0.05 FWE-corrected at the cluster level, with the height threshold at *p* < 0.005 uncorrected) [8].

#### 2.4.3. Functional Connectivity

Regions interact to maintain efficient emotional and cognitive functions. To investigate how sleep deprivation influences the interaction between regions in social feedback processing, we conducted a psychophysiological interaction (PPI) analysis using CONN to examine task-related connectivity of the insula as defined in the Harvard–Oxford subcortical structural atlas in FSL [21,22]. The insula was selected because it has been identified as a core region in both social-emotional and cognitive processing [8]. Furthermore, anatomical regions of interest were used as seeds to avoid any bias that may be introduced by using seeds derived from the current study [23]. Correction for multiple comparisons was performed using a voxel-level threshold of *p* < 0.001 and a cluster-level threshold of *p* < 0.05, FWE-corrected.

## 3. Results

### 3.1. First Impression Task of the Social Evaluation Task Experiment

The 78 participants were divided into two groups, normal sleepers (11 men of 37, PSQI ≤ 7 and ISI < 7) and poor sleepers (10 men of 41, PSQI > 7 or ISI ≥ 7) [19]. There were no differences in age (*t* = 0.427, *p* = 0.670) and sex (*χ*^2^ = 0.071, *p* = 0.790) between the groups. The mean age of the poor sleep group was 19.68, while for the normal sleep group, it was 19.54. Based on an independent samples *t*-test, participants with sleep disturbances did not evaluate others’ faces as less appealing (*t* = −1.86, *p* = 0.066), but were less willing to socialize with others (*t* = −2.19, *p* = 0.031) (Table 1). In the normal sleep group, the average rating for others’ facial appearance was 3.23 and the participants’ willingness to socialize was 3.50. In the poor sleep group, the average rating of others’ facial appearance was 2.88 and the participants’ willingness to socialize was 3.06. The two groups did not show any differences in levels of social anxiety (*t* = 0.825, *p* = 0.412) and rejection sensitivity (*t* = −0.087, *p* = 0.931). There was also no difference in the level of sleepiness (*t* = 0.206, *p* = 0.837).

### 3.2. Social Feedback Task of Social Evaluation Task Experiment

**Figure 2:**
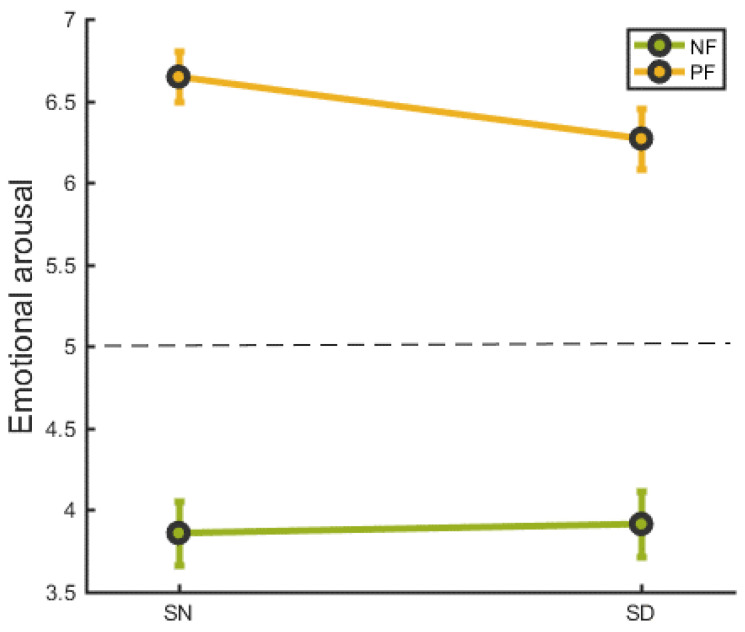
The emotional response following social feedback in SN and SD groups. NF = negative feedback, PF= positive feedback. SN refers to a night of normal sleep, SD refers to a night of deprivied sleep. Error bars indicate the standard error.

We conducted a repeated measures two-way analysis of emotional arousal based on sleep condition (SN vs. SD) and feedback type (negative vs. positive). As illustrated in Figure 2, the emotional ratings were, for positive feedback *M* ± *SD* = 6.65 ± 0.79, and, for negative feedback *M* ± *SD* = 3.86 ± 0.98, in the SN group. The emotional ratings were, for positive feedback *M* ± *SD* = 6.27 ± 0.93, and, for negative feedback *M* ± *SD* = 3.91 ± 1.02, in the SD group. There was no interaction effect between sleep and feedback condition (*F*(1,21) = 2.529, *p* = 0.127). 

#### 3.2.1. Whole-Brain Analysis

To assess differential whole-brain responses to two feedback conditions in each sleep state, we directly compared the response with negative feedback and positive feedback in the SN and SD conditions separately. As shown in Table 2, in response to negative feedback (vs. positive), the SN group showed less activation in the left superior frontal gyrus (SFG), left precentral gyrus, right postcentral gyrus, right SFG, left middle cingulate cortex, left inferior parietal gyrus, left cingulate cortex, and left precuneus. The SD group showed greater activation in the right inferior frontal gyrus (IFG) and cuneus.

**Figure 3:**
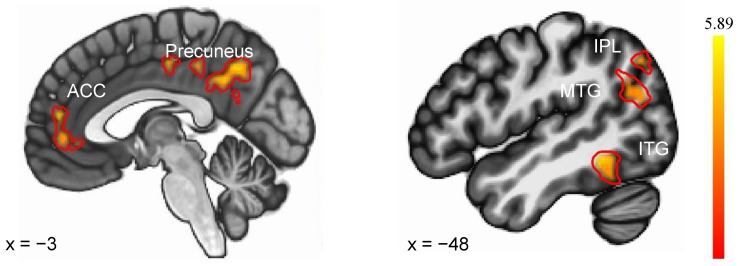
Brain regions showed differential responses to negative feedback (vs. positive feedback) between SN and SD. ACC—anterior cingulate cortex, IPL—Inferior Parietal Lobule, MTG—middle temporal gyrus, ITG—inferior temporal gyrus.

As illustrated in Figure 3, the evaluation of Sleep Condition × Feedback Condition interaction identified regions where the SN and SD groups displayed distinct differential responses to negative versus positive feedback. The SD (vs. SN) group exhibited an enhanced response to negative feedback versus positive feedback, localized to the left superior parietal lobule (SPL), left Inferior Parietal Lobule (IPL), left precuneus, left inferior temporal gyrus (ITG), left medial frontal gyrus (MFG), left and right anterior cingulate cortex (ACC), and left middle temporal gyrus (MTG). There were no areas of SN (NF vs. PF) > SD (NF vs. PF) activity. Details were shown in Table 3.

#### 3.2.2. gPPI Analysis

**Figure 4:**
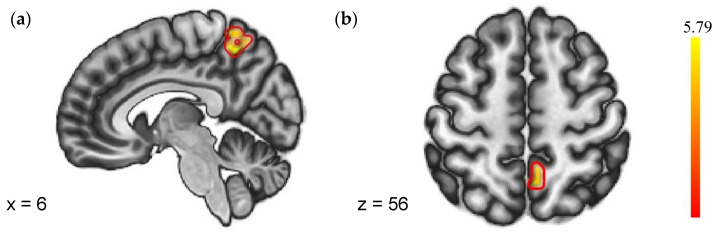
Regions with different FC to negative feedback (VS. positive feedback). (**a**) Sagittal plane with x = 6; (**b**) Axial plane with z = 56.

As shown in Figure 4, a group-level model of the PPI estimates revealed that SN (NF vs. PF) > SD (NF vs. PF) had significantly greater negative coupling from the right insula to the precuneus. No regions showed different FC with the left insula in the two sleep conditions. Details were shown in Table 4.

## 4. Discussion

We conducted a revised social evaluation task experiment to examine the relationship between sleep loss and social evaluation processing. The revised experiment consisted of a first impression task that required participants to give evaluations and a social feedback task that required participants to receive evaluations. We found that, while participants with sleep disturbances did not consider others as less attractive, they were less willing to socialize with others. The emotional response to social feedback showed no difference between SN and SD groups. However, brain regions including the SPL, precuneus, ITG, MFG, ACC, and MTG had greater activation to negative feedback after sleep deprivation. Most of these regions are parts of the default mode network [24]. In addition, co-activation of the insula–precuneus indicated stronger cognitive control during negative feedback processing after SD.

### 4.1. Negative Trend in Social Involvement 

We conducted a first impression task to improve the reliability of feedback received during fMRI scanning. We also investigated whether chronic sleep loss can lead to negative social evaluation of others. Consistent with the previous studies, those who sleep worse were less socially active [5,6]. This may be due to high fatigue followed by sleep disturbances [25]. However, such people did not consider others as less appealing. The reason why they did not want to be involved in social activities may not be associated with the evaluation of others’ attractiveness. The trustworthiness of others‘ faces may be more important than attractiveness in a social context [13]. 

In addition, participants had distinct brain activation patterns between normal sleep and deprived sleep with higher activation to positive feedback after a night of normal sleep and larger responses in relative regions to negative feedback after a night of sleep deprivation. The aberrant activation patterns after sleep deprivation demonstrated hyperactivation to negative feedback. Negative feedback was considered to be a sign of strained relationships. Therefore, neurophysiological responses followed by social rejection motivated us to adjust our social networks [10]. Because hyperarousal to negative feedback is harmful and related to several emotional disorders [26], participants may avoid engaging in social activities to prevent potential harm in a social situation.

### 4.2. Changes in Self-Referential Processes after Sleep Deprivation 

Participants were likely to commit more resources to negative self-related information processing after sleep deprivation. Relative activation in the default network as well as aberrant activation patterns may help explain this trend.

The temporal and parietal gyrus and precuneus have been identified as core regions of the DMN [24]. These regions supported self-related mental processes (e.g., monitoring external stimuli) and displayed a higher level of activity in the self-referential process [27]. Participants showed larger activation in the DMN when receiving negative feedback after sleep deprivation. The activation patterns showed that negative feedback was considered more important than positive feedback. However, positive feedback toom up more cognitive resources in healthy sleepers [9,14]. For healthy subjects, positive feedback was an important sign to guide their social behaviors. However, fighting against potential damage to self-concept was much more important after sleep deprivation. This is why there were no changes in self-reported arousal after SD, but greater activation in the precuneus and increased negative coupling of the precuneus and insula.

The Important role delegated to the insula is to detect, process, and react to salient events [28]. The precuneus is crucial for thinking about the self and whether information is relevant to the self [7]. Participants showed increased activation in the precuneus after SD, which may protect against potential damage from negative feedback. Meanwhile, the precuneus has a stronger negative FC with the insula, which may further reduce the emotional arousal triggered by negative feedback. 

Sleep deprivation has long been known to negatively affected self-regulation [29]. The aversive result found in our study may show that the impact of sleep deprivation is context-dependent. Negative feedback in this experimental context is related to self–other representation [8]. It can potentially damage self-value and was considered of great importance in a social context [11]. In order to avoid potential damage, healthy subjects automatically inhibited negative feelings induced by negative evaluations [30]. The automatic control process was almost unaffected by acute sleep deprivation [31], especially in a social context. This was considered to be a self-conservative mechanism. 

Even if the brain showed increased regulation ability for negative feedback, hyperactivation to negative feedback was still a warning sign for healthy social behaviors. Feedback rated as more applicable was related to increased activation in relative regions [7]. An alternative hypothesis is that greater brain activation for negative feedback may indicate a negative evaluation of themselves after SD. As participants in this study only reported their emotional arousal, determining whether greater activation in the DMN indicated greater negative self-view must be an object of future study. 

### 4.3. Limitations and Future Implications

Completing the social evaluation task twice may make it challenging to obtain reliable social feedback. So we designed a revised social evaluation task experiment with two important modifications to the original social evaluation task. The first is that participants have to complete an evaluation of facial images in order to increase the reliability of the feedback received. The second is that participants only need to receive negative or positive feedback during scanning, as we are only concerned with the valence of the feedback. Through this modified approach, we have increased the validity of the social feedback task and found the relationship between sleep status and social evaluation behaviors. However, there are two main drawbacks to the first impression task. Firstly, due to the time cost, we have not collected neural responses while the participants give evaluations, and the neurophysiological responses underlying how sleep-deprived sleepers evaluate others‘ faces is also an interesting question to investigate. Second, our sample size is relatively small, which weakens the certainty of the conclusions. 

In addition, in the social feedback task, we did not ask participants to report their subjective feelings about themselves. This is unfortunate, as our study showed functional impairments after SD mostly focused on regions involved in self-referential processing. The relationship between sleep loss and self-concept could be better determined by using both subjective reporting and neural imaging evidence.

Our study found that sleep loss may affect how we process ourselves. Damage to self-view is potentially harmful, as a negative self-view is a typical symptom of depression and anxiety disorders [32,33]. Our study may shed new light on how sleep loss leads to emotional disorders. In addition, the relative regions within the DMN can be target regions during neurofeedback. This is a powerful intervention method that can help patients improve their cognitive control abilities [34].

## 5. Conclusions

The results of the present study show that sleep loss alters our behavioral and neural responses to social evaluation. In terms of behavioral responses, participants reported less willingness to engage in social activities, which indicated a potential trend toward social withdrawal. In terms of neural responses, greater activation to negative feedback was observed in regions involved in self-referential processing after sleep deprivation. And stronger region coupling was found between the insula and precuneus. These changes in brain function may indicate an abnormal state upon processing information related to self-concept. The observed hyperactivation within the DMN indicated a negative bias to social feedback. And stronger FC indicated a conservational mechanism of self-concept after acute sleep deprivation. Our brain fights to protect against potential damage to self-concept. However, whether this effect exists in cases of chronic sleep loss remains unknown. We must keep in mind that the damage caused by sleep deprivation is profound.

## Figures and Tables

**Table 1:** Behavioral results of first impression task in poor and normal sleep groups.

	Poor Sleepers 10 M/41	Normal Sleepers 11 M/37	*t*	*p*
Age	19.68 ± 1.54	19.54 ± 1.35	0.427	0.670
KSS	4.74 ± 0.13	4.70 ± 0.15	0.206	0.837
Social anxiety	14.22 ± 4.93	13.30 ± 4.93	0.825	0.412
Rejection sensitivity	9.97 ± 2.61	10.03 ± 2.89	−0.087	0.931
Facial appearance	2.88 ± 0.89	3.23 ± 0.73	−1.864	0.066
Willingness to socialize	3.06 ± 0.95	3.50 ± 0.81	−2.194	0.031

**Table 2:** Results of negative feedback (NF) > positive feedback (PF) and PF > NF in normal sleep (SN) and deprived sleep (SD) conditions.

Region Labels	x	y	z	Cluster Size	*t*	*p* (ClusterFWE)
N: NF > PF						
None						
SN: PF > NF						
L Superior frontal gyrus	−14	40	36	179	7.85	<0.001
L Precentral gyrus	−28	−22	56	471	6.46	<0.001
R Postcentral gyrus	48	−32	40	127	6.47	<0.001
R Superior frontal gyrus	18	32	44	151	5.92	<0.001
L Middle cingulate cortex	−2	−34	42	221	5.89	<0.001
L Inferior parietal gyrus	−50	−38	56	102	5.95	<0.001
L Inferior parietal gyrus	−50	−70	42	88	5.38	<0.001
L Cingulate cortex	−2	−34	42	233	5.91	<0.001
L Precuneus	−4	−60	42	87	5.34	<0.001
SD: NF > PF						
R Inferior frontal gyrus	48	24	−2	292	4.86	<0.005
R Cuneus	14	−70	18	244	4.49	<0.005
SD: PF > NF						
None						

**Table 3:** Regions showing significant activity between normal sleep (SN) and deprived sleep (SD) (negative feedback > positive feedback).

Region Labels	x	y	z	Cluster Size	*t*
SN > SD					
None					
SD > SN					
L Superior Parietal Lobule	−34	−76	46	266	5.89
L Inferior Parietal Lobule	−46	−70	42		
L Precuneus	−6	−58	44	1409	5.11
L Inferior temporal gyrus	−48	−48	−10	751	4.93
L Medial frontal gyrus	−6	52	14	314	4.74
L Anterior cingulate	−4	38	−4		
R Anterior cingulate	6	44	0		
L Middle temporal gyrus	−38	−66	22	283	4.08

**Table 4:** Regions showing significant FC with insula in whole brain.

Region Labels	x	y	z	Cluster Size	*t*
Seed: R insula					
precuneus	6	−52	54	177	5.79
Seed: L insula					
None					

## Data Availability

The data associated with the paper are not publicly available but remain available from the corresponding author upon reasonable request.
